# Spatiotemporal trends of carbon stock in wood and bamboo products in China during 1987–2020

**DOI:** 10.1038/s41598-023-41007-6

**Published:** 2023-09-14

**Authors:** Luyang Zhang, Shuaikun Zhang, Dongdong Chen, Tianrun Li, Shiyi Wang, Zhiyuan Xu, Yuchao Wang, Zhihua Liu, Shaoliang Zhang

**Affiliations:** 1 School of Resources and Environment, Northeast Agricultural University, No.600 Changjiang Road, Xiangfang District, Harbin, 150030 China; 2 Key Laboratory of Germplasm Innovation and Physiological Ecology of Crops in Cold Regions, Ministry of Education, Harbin, China; 3 Joint Laboratory of Agriculture coping with Climate Change of China Meteorological Administration (CMA) and China Agricultural University (CAU), Beijing, China; 4 The Agrometeorological Center of Sichuan Province/Provincial Key Laboratory of Water-Saving Agriculture in Hill Areas of Southern China, Chengdu, 610072 China

**Keywords:** Climate change, Forestry

## Abstract

Harvested wood/bamboo products (HWP/HBP) constitute a large global carbon stock. However, the contribution of HBP to carbon stocks has been neglected in mixed wood and bamboo data, especially in China. Therefore, the production approach and the first-order decay method were used to estimate the spatiotemporal carbon stock change in HWP/HBP based on provincial production data from the China Forestry Statistical Yearbooks for 1987–2020. The results showed that China’s total carbon stocks of HWP and HBP were 328.7 teragram carbon (TgC) and 129.7 TgC between 1987 and 2020. Of this, the HWP carbon stock was mainly sourced from three provinces across the north and south: Guangxi (60.8 TgC), Heilongjiang (37.2 TgC), and Fujian (24.2 TgC), and HBP carbon stock was mainly sourced from three southern provinces: Fujian (33.4 TgC), Guangxi (20.3 TgC), and Zhejiang (13.7 TgC). The proportion of the HBP carbon stock in the total carbon stock increased from 20% in 2010 to 28% in 2020, indicating that bamboo products play an important role in the accumulation of carbon stocks in China. The differences in contributions to spatiotemporal trends between the provinces provide more specific information to make precise decisions about forest management and carbon sequestration.

## Introduction

Forests are crucial to the global carbon cycle; they absorb 7.6 ± 49 billion tons CO_2_ equivalent per year, up to 30% of annual global anthropogenic CO_2_ emissions^1,2^. Carbon stored in harvested wood products (HWP) plays a mitigating role as a commodity. In 2020, global roundwood production was approximately 3.9 billion m^3^, an increase of 25% compared to volumes in 1980^3^. With the growing use of wood products worldwide, the global HWP pool sequestered 335 million tons (Mt) of CO_2_ equivalent per year in 2015 and is predicted to increase to 441 Mt of CO_2_ equivalent per year by 2030^4,5^. This indicates that increasing the use of HWP, especially long-lived products produced from sustainably managed forests, could delay the release of CO_2_ into the atmosphere, thus allowing more time to address climate change.

As China is one of the major producers and consumers globally^6^, the carbon stock of HWP in China is a notable carbon sink^7–12^. The stock-change approach can reveal all carbon stocks of forest products consumed by the reporting country, regardless of where the forests are harvested^4^. Using the stock-change approach and data from the Food and Agriculture Organization Statistical databases, FAOSTAT, the carbon stock of China reached 680.64 teragram carbon (TgC) from 1900 to 2015^8^. An improved stock-change approach using Eora multiregional input–output tables linked to global HWP production and consumption estimated that the carbon stock of in-use HWP in China during 1992–2015 was 480 TgC^11^. Zhang et al.^9^ also estimated the HWP consumed in China using a stock-change approach, but obtained a more comprehensive scope of HWP, including in-use HWP, discarded mill residue, wood fiber input, and waste HWP from the China Forestry Statistical Yearbooks. They evaluated the carbon stock in China from 1900 to 2015 to be 2195.2 TgC. The carbon stock of forest products produced in China can be assessed using a production approach, wherein imported forest products are excluded from the reporting country^4^. Using a production approach, the carbon stocks of in-use HWP and HWP deposed at solid waste disposal sites during 1900–2016 were 649.2 TgC and 72.6 TgC, respectively^10^. Using a refined production approach^13^, Yu et al.^12^ estimated that the in-use HWP carbon stock until 2019 was 815.94 TgC. However, the previous studies focused on the scale of the country; they indicated dissatisfaction with the recommendations of the forestry management policy, which should be based on more precise regional data, for example, at the provincial scale. Starting in 2000, the Natural Forest Protection Project (NFPP) was implemented in 17 provinces to mitigate environmental damage^14^. The NFPP has since covered 37% of the country's land area, including the establishment of protected areas, replantation of trees, and setting up of animal rescue centers^15^. These policies had a clear impact on timber harvesting, and each province faced implementation scenarios. Understanding the provincial carbon stock accumulation also reflects the effectiveness of the NFPP policy.

As the FAOSTAT database does not clearly distinguish between harvested wood and bamboo products^16^, the HWP carbon stock in China, estimated using mixed wood and bamboo data^8–11^, reduces the reliability of the carbon stock from harvested bamboo products (HBP). China has the richest bamboo resources worldwide, accounting for 18% of all bamboo forests^17,18^. At the end of 2020, the output value of the production of China's bamboo industry reached 319.9 billion CNY^19^. Thus, HBP contributes greatly to carbon stocks, such as Moso bamboo products that store 10.19 ± 2.54 TgC per year^20^. However, when estimating the HWP carbon pool, it remains unclear how much of the carbon stock originates from bamboo^7,8,10–12^. Recently, bamboo production data from the China Forestry Statistical Yearbooks were used to assess HBP carbon stocks^20–22^. HBP carbon storage has increased from 9.67 to 55.33 TgC during 1993–2018 in China^22^, showing a sizable gap when compared with 199.07 TgC for the HBP carbon stock in 2000–2009^21^. Therefore, attention is needed to determine the contribution of bamboo products to carbon stocks.

In this study, we used the production approach and first-order decay (FOD) method as standard models to simulate the carbon decay of forest harvesting, as recommended by the Intergovernmental Panel on Climate Change (IPCC)^23,24^. Provincial HWP and HWP/HBP production statistics from the China Forestry Statistical Yearbooks were used to estimate the characteristics and changes in carbon stocks in China from 1987 to 2020. The objectives of this study were to: (1) present spatiotemporal changes in the carbon stock of HWP/HBP at the provincial scale in China and (2) compare the contribution proportion of the carbon stock from HWP and HBP in China. In this study, we proposed two hypotheses. First, we hypothesized that there are large differences in carbon stock in China at the provincial scale because the country has a large land area and the provinces are distributed across areas of different meteorological conditions^25^. Over the past 40 years, the bamboo forest area in China has increased by an average of 81,300 ha per year^18^, reaching 7.53 million ha by 2021. Of this area, 67.47% comprises commercial bamboo forests. Management tactics such as fertilization within the managed bamboo forests has also increased yields^26^. Therefore, our second hypothesis considers that the importance of carbon stock from HBP has increased over time.

## Methods

To estimate the carbon stocks of HWP/HBP produced in China using the production approach, we obtained provincial HWP/HBP production data from the China Forestry Statistical Yearbooks from 1987 to 2020^27^. We collected data on the production of roundwood, roundwood for paper products, and fuelwood from 1987 to 2020; Moso bamboo (*Phyllostachys edulis*)^22^ and clumping bamboo (*Bambusa pervariabilis*)^28^ from 1992 to 2020; and other small-diameter bamboo from 2003 to 2020 in each province of China (without data for Hong Kong, Macau, and Taiwan). Some inconsistency occurred within the calculated years because of data collection initiating in different years. According to the China Forestry Statistical Yearbooks, all provinces claimed HWP production to varying degrees. Bamboo forests mainly grow in subtropical southern China^29^; thus, 20 provinces in that region counted bamboo products: Fujian, Jiangxi, Zhejiang, Hunan, Sichuan, Guangdong, Guangxi, Anhui, Guizhou, Hubei, Hunan, Hainan, Chongqing, Yunnan, Henan, Shanghai, Gansu, Shaanxi, Shandong, and Tibet. For this study, it was assumed that carbon in the primary HWP/HBP was transferred to the finished products and that carbon in the fuelwood was released within the year of data collection.

### Annual carbon inflow to the HWP/HBP pool

HWP was divided into roundwood for paper products, industrial roundwood, and fuelwood based on data of primary wood products in China. The annual carbon inflows of paper products and industrial roundwood were calculated using Eqs. (1) and (2):

1 $${C}_{HWP,i}={H}_{i}\cdot {C}_{f1}$$

2 $${H}_{i}={S}_{i}+{P}_{i}-{F}_{i}$$

${C}_{HWP,i}$: carbon content of HWP of year *i*.

*H*_*i*_: the production of HWP of year i, m^3^.

${S}_{i}$: Production of industrial roundwood in year i, m^3^.

${P}_{i}$: Roundwood production for paper products in year i, m^3^.

${F}_{i}$: fuelwood production in year i, m^3^.

${C}_{f1}$: carbon factor to convert roundwood from volume units to carbon units, tons C m^−3^, which was sourced from the recommendations of the IPCC^23^ as shown in Table 1.

**Table 1:** Carbon convention factors of HWP and HBP.

	Roundwood, fuelwood		Moso bamboo	Clumping bamboo	Small-diameter bamboo
Density (oven-dry tonne m^−3^)	0.52*	Biomass per culm (kg)	23.69	10.611	–
Carbon fraction	0.5		0.53	0.5	0.45
		Moisture content	0.452	0.44	0.43
Carbon convention factor	${C}_{f1}=$ 0.26 (t C m^−3^)		${C}_{f2}=$ 6.86 (kg culm^−1^)	${C}_{f3}=$ 2.97 (kg culm^−1^)	${C}_{f4}=$ 0.26 (t C t^−1^)

*Half temperate species and half tropical species were assumed.

The annual carbon inflow of bamboo was calculated using Eq. (3):

3 $${C}_{HBP,i}={B}_{1,i}+{B}_{2,i}+{B}_{3,i}={n}_{1}\cdot {C}_{f2}+{n}_{2}\cdot {C}_{f3}+W\cdot {C}_{f4}$$

${C}_{HBP,i}$: carbon content of HBP of year *i*.

*B*_*i*_ is the production of different types of bamboo (*Phyllostachys pubescens*, i.e., Moso bamboo, clumping bamboo, and other small-diameter bamboo) in year i; *n*_*1*_ is the number of harvested Moso bamboo;* n*_*2*_ is the number of clumping bamboo; and *W* is the weight of all other small-diameter bamboo tons.

${C}_{f2-4}$: carbon factor to convert Moso bamboo, clumping bamboo, and other types of small-diameter bamboo from volume units to carbon units, as shown in Table 1.

The biomass per culm and moisture content of Moso bamboo were determined following Shi et al.^30^, and the carbon fraction of Moso bamboo was obtained from Liu et al.^31^. The biomass per culm and moisture content of clumping bamboo followed the studies of Huang^32^ and Ou^33^, and applied the carbon fraction from Tian et al.^34^ and Qin et al.^35^. The carbon fractions and moisture contents of other types of small-diameter bamboo were obtained from Lun et al.^21^ and Guo^36^.

### Annual change of carbon stock in HWP/HBP

FOD was used to estimate carbon change in HWP/HBP. The estimation of changes in carbon stocks in HWP/HBP was obtained using Eqs. (4)–(6):

4 $$C\left(i+1\right)={e}^{-k}\cdot C\left(i\right)+[\frac{(1-{e}^{-k})}{k}]\cdot Inflow(i)$$

5 $$\Delta C\left(i\right)=C\left(i+1\right)-C\left(i\right)$$

6 $$Inflow\left(i\right)={C}_{HWP,i}+{C}_{HBP,i}$$

*C(i)* is the carbon stock at the beginning of year *i* and *C* (1987) = 0. The HWP/HBP produced before 1987 was not considered in this study. *k* = *ln (2)/HL*, where *k* represents the decay constant of FOD and is given in units, yr^−1^. *HL* is the half-life of the HWP pool in years.

**Figure 1:**
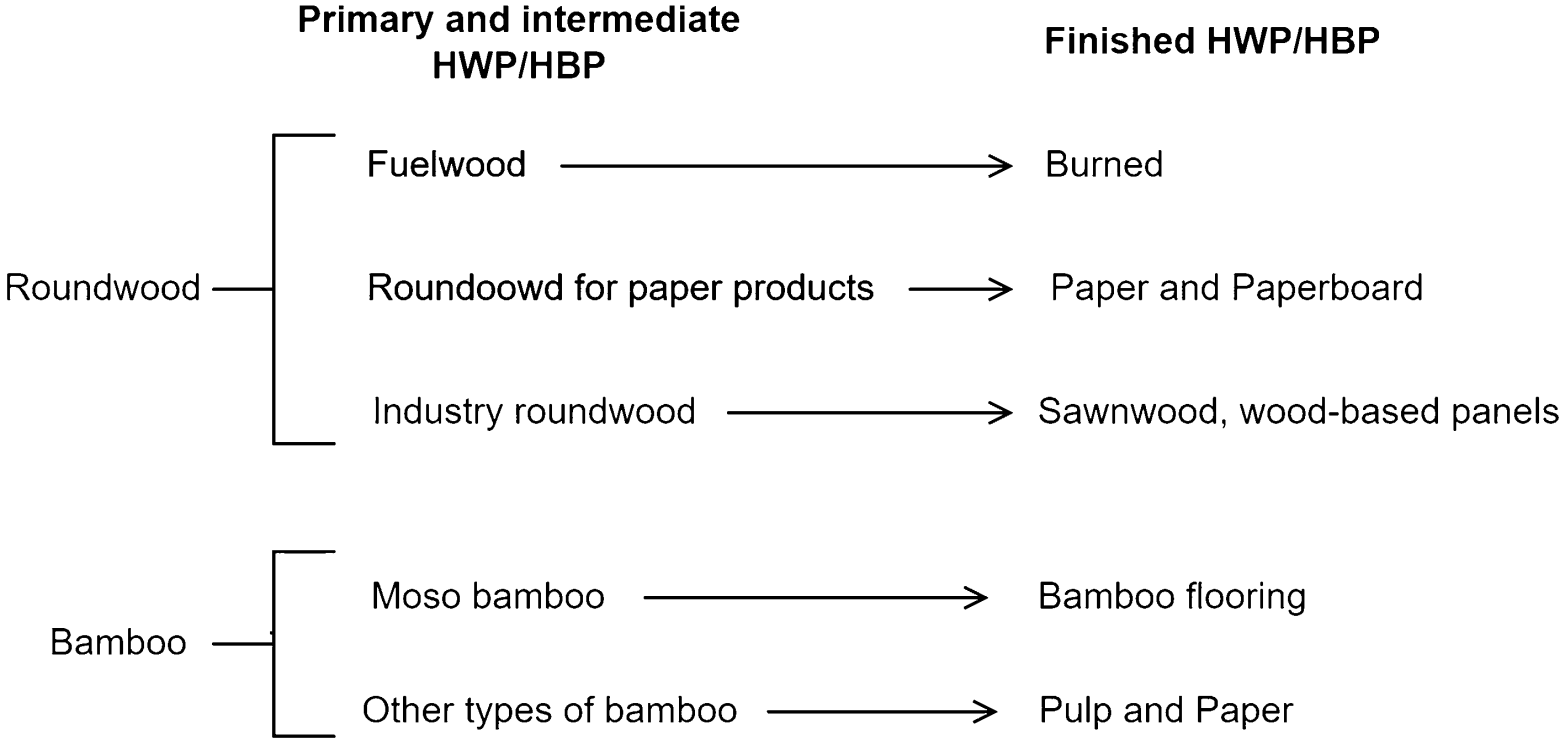
Classification of HWP/HBP.

The half-lives of wood-based panels and sawn wood are 25 and 35 years, respectively^24^. We assumed 30 years of industrial roundwood to be the average for wood-based panels and sawn wood. The half-life of roundwood for paper products is 2 years^24^. Moso bamboo has a diameter of approximately 10–12 cm, is typically used as a timber material for flooring and construction^37,38^, and has a half-life of 10 years^39^. Clumping bamboo and small-diameter bamboo are mainly used in the pulp and paper industry; therefore, we set the half-life of 2 years to match that of roundwood for paper products. The HWP/HBP classification is shown in Fig. 1.

## Results

### Annual carbon inflow in HWP/HBP

**Figure 2:**
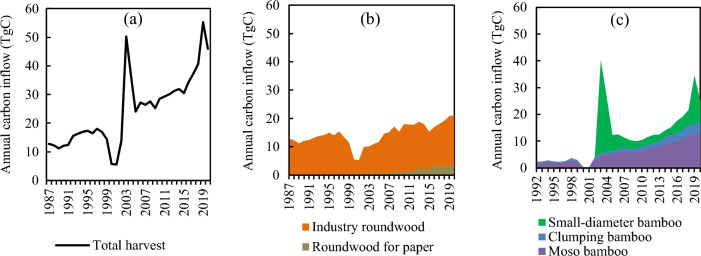
Annual carbon inflow of (**a**) total harvest HWP and HBP from 1987 to 2020, (**b**) HWPs from 1987 to 2020, and (**c**) HBPs from 1992 to 2020.

The total carbon inflow of the HWP/HBP roughly quadrupled from 1987 to 2020, and sharp fluctuations occurred between 2000 and 2005 (Fig. 2a). The smallest carbon inflow of HWP/HBP was 5.6 TgC in 2001, and the largest was 55.3 TgC in 2019. The carbon inflow of HWP increased from 12.8 TgC in 1987 to 21.1 TgC in 2020 with dramatic declines in 2000 (down to 5.5 TgC) and 2001 (5.3 TgC). Among HWPs, the carbon inflow of industry roundwood increased from 12.4 TgC in 1987 to 17.9 TgC in 2020, which dominated the HWP carbon inflow, accounting for 85% of the total in 2020 (Fig. 2b). The carbon inflow of HBP increased from 2.3 TgC in 1992 to 24.8 TgC in 2020, with two peaks of 40.1 TgC in 2003 and 34.5 TgC in 2019, showing a more volatile upward trend compared to HWP (Fig. 2c). Among HBPs, the carbon inflow of Moso bamboo grew steadily from 1.9 to 13.0 TgC and that of clumping bamboo increased from 0.38 to 4.0 TgC during 1992–2020. In contrast, the carbon inflow of small-diameter bamboo was unstable, showing high carbon inflows in 2003 (34.6 TgC) and 2004 (20.2 TgC); furthermore, the average carbon inflow was only 3.7 TgC during 2005–2018. Carbon inflow from small-diameter bamboo increased again in 2019 with 18.0 TgC, but dropped to 7.9 TgC in 2020. Two peaks of total carbon inflow of HWP/HBP (50.2 TgC in 2003 and 55.3 TgC in 2019) corresponded to the increase of small-diameter bamboo, which accounted for 69% of total carbon inflow in 2003 and 33% in 2019. This percentage was mostly below 20% in the other years.

### Carbon stock in HWP/HBP

**Figure 3:**
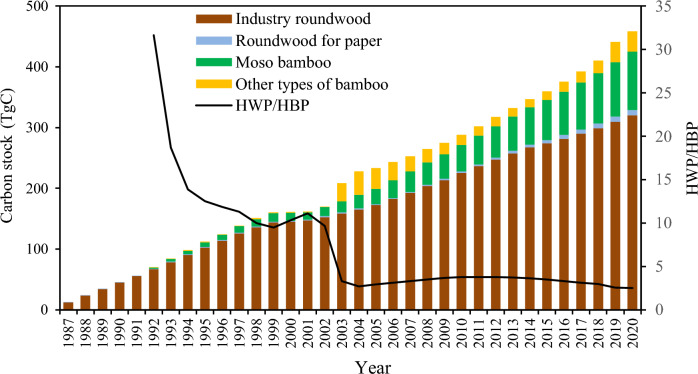
Change in carbon stock in HWP/HBP from 1987 to 2020.

The carbon stock represented sustained growth in the period of 1987–2020 in China, and was estimated to be 458.5 TgC, of which the carbon stock from HWP and HBP was 328.7 and 129.7 TgC, respectively (Fig. 3). In 2020, industrial roundwood contributed to most carbon stocks (320.3 TgC), followed by Moso bamboo (96.2 TgC). Carbon stocks of other types of bamboo and roundwood for paper accounted for only 7% and 1.8% of the total, respectively, at the end of 2020. The proportion of carbon stocks in HWP reduced dramatically compared to that in HBP from 1992 to 2020; in 1992, the carbon stock of HWP was 31.6 times greater than that of HBP, while this ratio fell to 2.5 times in 2020.

### Spatiotemporal analysis of carbon stock in HWP/HBP

**Figure 4:**
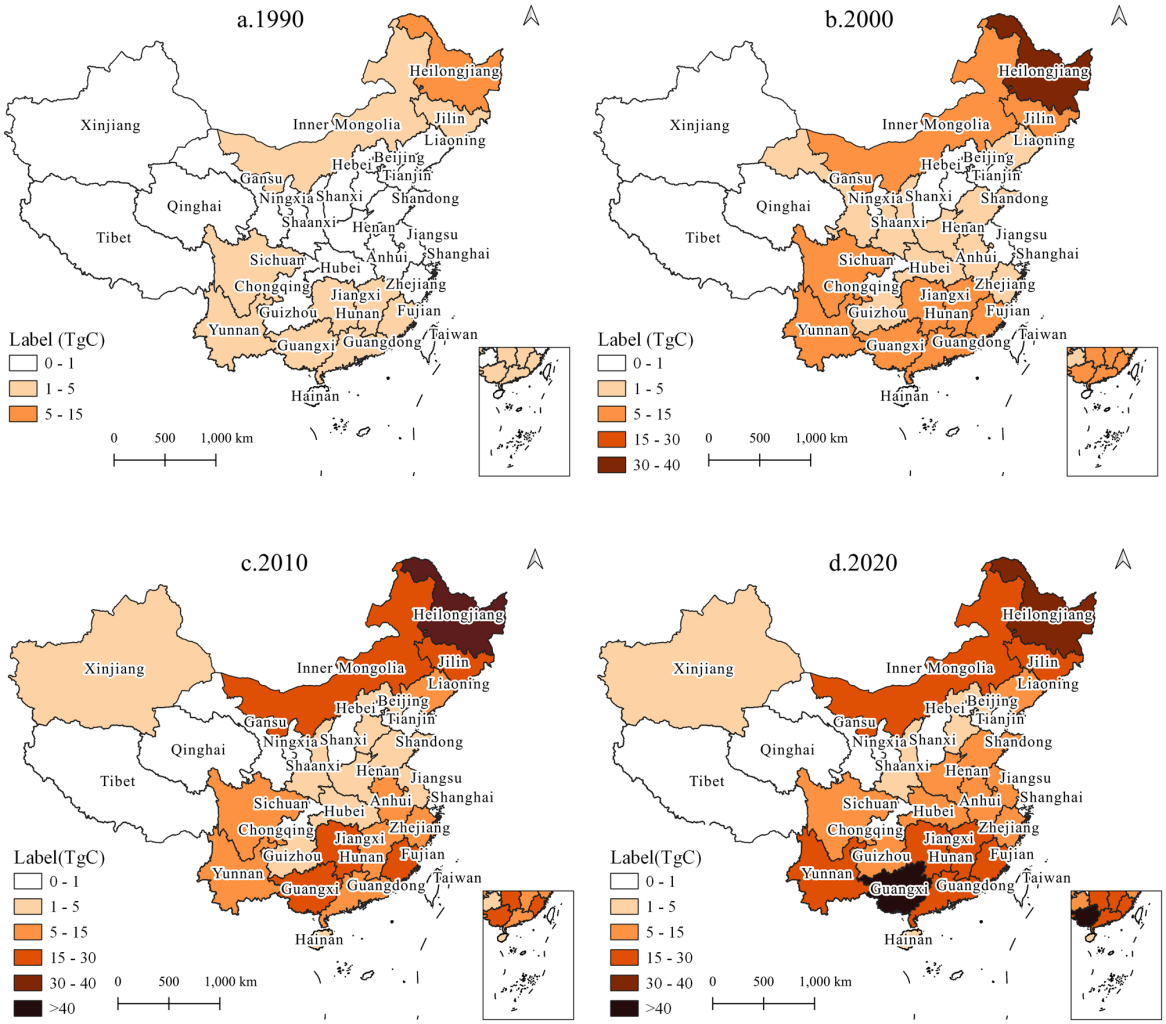
Spatiotemporal evolution of HWP carbon stock in China's provinces.

**Figure 5:**
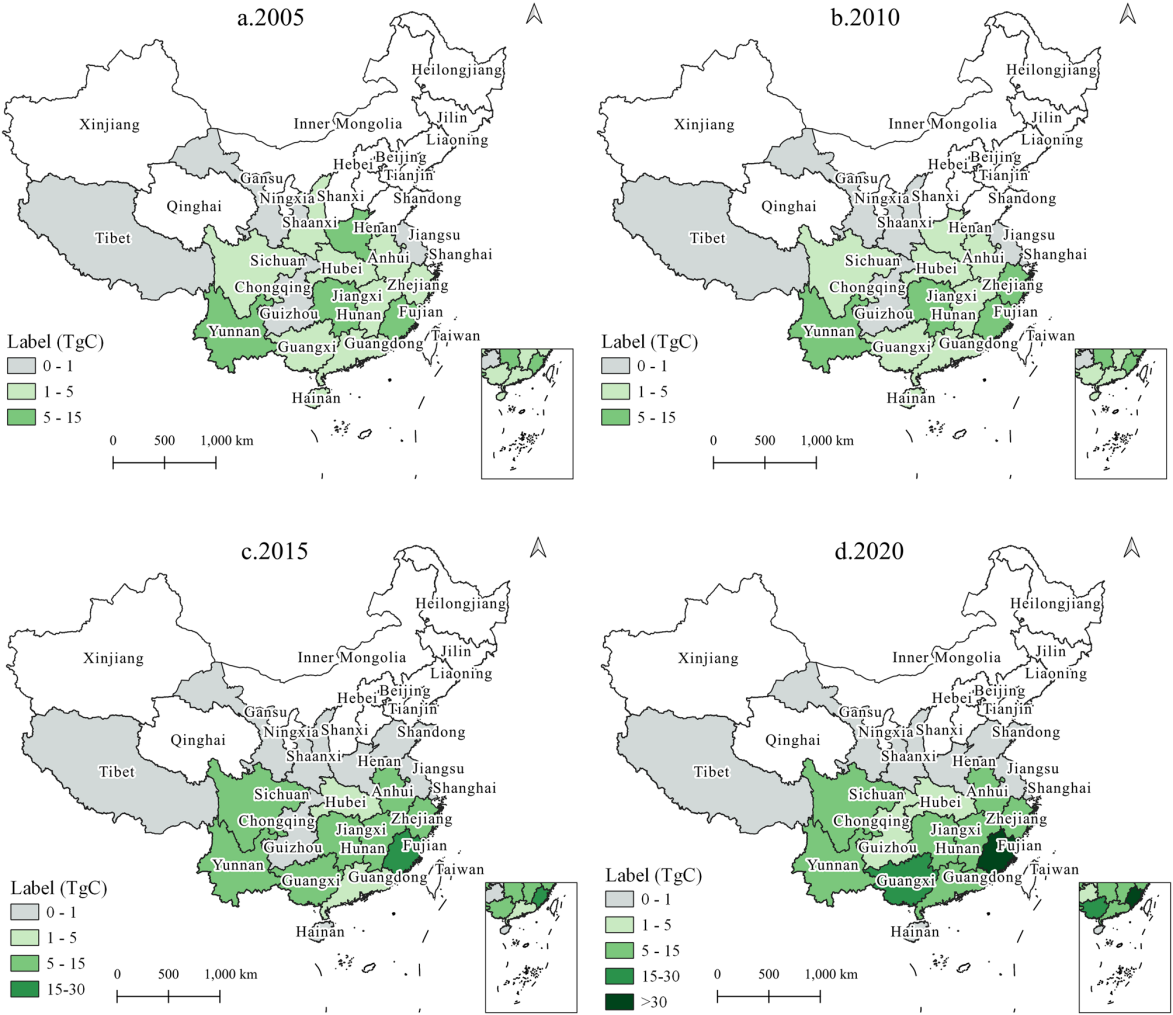
Spatiotemporal evolution of HBP carbon stock in China's provinces.

Spatial variations in carbon stocks were identified among the regions of China (Figs. 4 and 5). The main contribution to HWP carbon stock originated from provinces in northeastern and southern China (Fig. 4). At the end of 2020, the HWP carbon stock in Guangxi was 60.8 TgC, much higher than that in other provinces (Table 2). The top 10 contributing provinces—Guangxi, Heilongjiang, Fujian, Jilin, Guangdong, Hunan, Yunnan, Inner Mongolia, Jiangxi, and Anhui—accounted for 76% of China’s HWP carbon stocks (Table 2). Of these top 10 provinces, Heilongjiang, Jilin, and Inner Mongolia are located in the northeast, whereas the other provinces are located in the south. Xinjiang, Shaanxi, Gansu, Tibet, Qinghai, and Ningxia in western China represent the lowest carbon stocks (HWP carbon stock < 5 TgC). Other provinces with low HWP carbon stocks include Hebei, Shanxi, Beijing, and Tianjin in the north and Hainan, Chongqing, and Shanghai in the south. From a temporal perspective, Heilongjiang is the only province that showed obvious fluctuation, in which the HWP carbon stock reached a peak of 42.0 TgC around 2010 (the highest in the country) and then dropped to 37.2 TgC in 2020. The HWP carbon stock remained in a low state during 1987–2020, with little change in the western provinces (Tibet, Shaanxi, Gansu, Qinghai, and Ningxia) and municipalities (Beijing, Shanghai, Tianjin, and Chongqing). The HWP carbon stocks in all other provinces showed sustained growth from 1987 to 2020.

**Table 2:** Carbon stock of HWP/HBP in China's provinces during 1987–2020.

Province	Carbon stock of HWP (TgC)	Carbon stock of HBP (TgC)*	HWP/HBP	Total carbon stock (TgC)	Percentage of total carbon stock in China (%)
Guangxi	60.8	20.3	3.0/1	81.1	17.7
Fujian	24.2	33.4	0.7/1	57.7	12.6
Heilongjiang	37.2	–	–	37.2	8.1
Hunan	19.5	10	2.0/1	29.5	6.4
Guangdong	21.2	7.6	2.8/1	28.8	6.3
Yunnan	16.6	9.9	1.7/1	26.5	5.8
Jiangxi	15.3	10.9	1.4/1	26.3	5.7
Anhui	14.2	9.6	1.5/1	23.8	5.2
Jilin	22.5	–	–	22.5	4.9
Zhejiang	8	13.7	0.6/1	21.7	4.7
Sichuan	12	7.2	1.7/1	19.2	4.2
Inner Mongolia	16.3	–	–	16.3	3.5
Shandong	11	0.00002	550,000.0/1	11	2.4
Henan	8.6	0.2	43.0/1	8.8	1.9
Guizhou	7.3	1.5	4.9/1	8.8	1.9
Hubei	6.4	2.3	2.8/1	8.6	1.9
Liaoning	7.7	–	–	7.7	1.7
Jiangsu	5	0.4	12.5/1	5.4	1.2
Hainan	3.6	0.3	12.0/1	4	0.9
Chongqing	0.9	2.3	0.4/1	3.1	0.7
Hebei	2.7	–	–	2.7	0.6
Xinjiang	2.1	–	–	2.1	0.5
Shaanxi	1.6	0.5	3.2/1	2.1	0.5
Gansu	0.8	0.0063	127.0/1	0.8	0.2
Shanxi	0.7	–	–	0.7	0.2
Beijing	0.5	–	–	0.5	0.1
Tianjin	0.5	–	–	0.5	0.1
Tibet	0.5	0.0123	40.7/1	0.5	0.1
Qinghai	0.2	–	–	0.2	0
Ningxia	0.2	–	–	0.2	0
Shanghai	0.0024	0.00174	1.4/1	0.004	0

*Twenty provinces counted bamboo products in the China Forestry Statistical Yearbooks. Carbon stocks in provinces with no bamboo products are indicated by "–".

Twenty provinces counted bamboo products in the China Forestry Statistical Yearbooks (Fig. 5, Table 2). Among them, Tibet, Gansu, Shanghai, and Shandong had the lowest HBP carbon stock; the total HBP carbon stock of the four provinces was only 0.02 TgC until 2020 (Table 2). At the end of 2020, the provinces with the top three HBP carbon stocks were Fujian (33.4 TgC), Guangxi (20.3 TgC), and Zhejiang (13.7 TgC), accounting for 52% of the total HBP carbon stock. During 1992–2020, the HBP carbon stocks continued to grow in most provinces, including Fujian, Guangxi, Zhejiang, Jiangxi, Yunnan, Sichuan, Chongqing, Guizhou, and Jiangsu. The three provinces with the highest increases during 2005–2020 were Fujian (27.8 TgC), Guangxi (18.0 TgC), and Zhejiang (8.9 TgC). The HBP carbon stocks in Hunan, Anhui, and Guangdong showed a slight decrease between 2010 and 2015 and then resumed growth. In four provinces, Henan, Shaanxi, Hubei, and Hainan, HBP carbon stock peaked in 2005 at 6.0, 3.1, 4.2, and 3.7 TgC, but dropped to 0.2, 0.5, 2.3, and 0.3 TgC, respectively, in 2020.

The proportion of HBP carbon stocks in China increased from 20% in 2010 to 28% in 2020. In Fujian, Zhejiang, and Chongqing, the proportion of HBP carbon stocks in 2020 increased to 58.0%, 63.2%, and 72%, respectively. Although the proportions of HBP carbon stock in Guangxi and Guizhou were not as high in 2020, at 25% and 18%, respectively, they have shown continuous increase. The proportion of HBP carbon stocks in Jiangxi, Hunan, Yunnan, Anhui, Guangdong, and Sichuan fluctuated between 25 and 44% between 2005 and 2020. The proportion of carbon stocks in Hubei, Jiangsu, Hainan, Henan, and Shaanxi steadily declined between 2005 and 2020.

From the viewpoint of total HWP and HBP carbon stock, the top 10 contributing provinces—Guangxi, Fujian, Heilongjiang, Hunan, Guangdong, Yunnan, Jiangxi, Anhui, Jilin, and Zhejiang—represented 77% of China’s HWP/HBP carbon stock (Table 2). Eight provinces had contributions from HWP and HBP, except for Heilongjiang and Jilin, which had only HWP carbon stocks.

## Discussion

A large difference was found in the carbon stock, including total, HWP, and HBP, at the provincial scale in China during 1987–2020 (Figs. 4 and 5), supporting our first hypothesis. This uneven spatial distribution of carbon stocks was also observed in previous studies^21,22^. Previously, the top contributors to HWP carbon stock were Guangxi, Fujian, Heilongjiang, Hunan, and Jilin during 2000–2009^21^, which is consistent with the results of this study. The HBP carbon stock in each province was 4–5 times larger than in this study, although we obtained a similar provincial HBP carbon stock rank. Lun et al.^21^ claimed that the HBP carbon stock was 1.7 times larger than the HWP carbon during 2000–2009 in China, whereas the present study found that the HBP carbon stock accounted for 62% of the HWP carbon stock in the same period. This large difference is attributed to the assumption that 40% of the retired HWP/HBP was disposed in landfills; this assumption does not apply to the current situation. In this study, the fate of retired forest products was not considered due to a lack of data on wasted HWP/HBP. Furthermore, Lun et al.^21^ assumed that the biomass per culm of Moso bamboo was 63.46 kg, which is 2.7 times larger than in this study, and also larger than in most literature^30,40–42^. The study ignored the moisture content, eventually resulting in a larger HBP carbon stock. A similar situation was observed in the study by Zhang et al.^22^, because they used the same method and parameters from Lun et al.^21^ to estimate the HBP carbon stock. In China, HBP carbon storage of HBP plays an important role, but it would be an exaggeration to find that its carbon sequestration capacity has exceeded that of HWP. The carbon stock of our results (359.7 TgC) was similar to that estimated by Yang and Zhang^8^ for the same period 1987–2015, which was approximately 360 TgC; the methodological approach of each study was the same, using FOD and production data. The average annual carbon inflow of bamboo during 2004–2008 was 11.7 TgC, which was similar to the estimation by Li et al.^20^ of 10.19 ± 2.54 TgC per year during the same period.

The HWP carbon stock of China showed a steady increase over the period 1987–2020, with only trace increases in 2000 and 2001 (Fig. 3). This minimal carbon stock accumulation was caused by the dramatic decline in carbon inflow in 2000 and 2001 (Fig. 2), owing to China having suffered a severe drought during that period. In 2000, the drought impacted area occupied approximately 40.54 × 10^4^ km^2^, the largest measured drought area since 1951^43,44^. The severity of the drought continued into the following year; both north and south China were affected^43^. As a result, carbon inflows from both wood and bamboo were reduced overall. This demonstrates that although forest resources are the basis for timber production, carbon stocks do not fully correspond to these resources. The volume of forests differs across provinces and is mainly distributed in northeast and southwest China^45^. According to the Ninth National Forest Resources Inventory (2014–2018), Tibet, Yunnan, Sichuan, Heilongjiang, and Inner Mongolia had the top five forest stock volumes^45^, whereas the HWP carbon stocks until 2020 were 0.5, 16.6, 12.0, 37.2, and 16.3 TgC, respectively. In addition, forest management policies have affected the uneven development of the accumulation of provincial carbon stocks. The NFPP was implemented in 2000 to protect natural forests in Yunnan, Sichuan, Guizhou, Chongqing, Hubei, Tibet, Shaanxi, Gansu, Qinghai, Ningxia, Inner Mongolia, Shanxi, Henan, Inner Mongolia, Jilin, Heilongjiang, Hainan, and Xinjiang^15^. Owing to the NFPP, Heilongjiang changed its function from a major timber producer to a reservoir; therefore, its carbon stock declined after 2010 (Fig. 4). Tibet ranked first in natural forests, and also played a major ecological role. In Tibet, forest tourism and the collection of plateau medicinal herbs are the leading forestry economy, but the region has no timber harvesting for commercial purposes^46^. Therefore, there was little carbon stock from the HWP. Likewise, in Inner Mongolia, Sichuan, and Yunnan, which also have abundant natural forests as protected areas, their HWP carbon stock contributions did not correspond to forest resources due to logging restrictions.

However, bamboo forest area only accounted for 2.9% of China’s total forest area^18^, yet HBP contributed 28% to the total carbon stock. The carbon inflows of Moso bamboo and clumping bamboo showed a steady increase from 1992 to 2020, except for a sharp drop in carbon inflows of bamboo due to the severe droughts in 2000 and 2001^43,44^ (Fig. 2). In contrast, the carbon inflow of small-diameter bamboo fluctuated twice, during 2003–2004 and in 2019 (Fig. 2). The sharp increase in carbon inflow resulted from the large-scale harvest of small-diameter bamboo in Henan, Hunan, and Shaanxi during 2003–2004, and in Guangxi in 2019. Bamboo grows faster than its wood equivalents and there are no logging restrictions on bamboo^47^; hence, the HBP carbon stocks were proportional to bamboo forest resources (Fig. 5). Bamboo grows primarily in Fujian, Jiangxi, Zhejiang, Hunan, Sichuan, Guangdong, Anhui, Guangxi, Hubei, and Guizhou^18^, which provide 90% of the HBP carbon stock (Table 2). The heterogeneous distribution of bamboo forests resulted in a difference in the HBP carbon stocks between provinces. Most bamboo needs a warm and humid climate to grow^29^, while the average winter temperature in the area north of the Qinling Mountain-Huai River line is below 0℃^48^; therefore, bamboo resources are largely absent from the northern provinces. The Chinese government established subsidies to encourage bamboo cultivation and made efforts to promote the means to improve the pest and disease control in bamboo forests^49^. The development of the bamboo industry is an important means of promoting local income. China's bamboo products are sold to more than 20 countries and regions worldwide, with an export trade of around US$2.2 billion in 2020^49,50^. With the growth of bamboo forests and increase of yield, the HBP carbon stock in China doubled between 20,005 and 2020. The proportion of HBP carbon stock in the total carbon stock also increased from 20% in 2010 to 28% in 2020, supporting our second hypothesis.

A few limitations exist in the present study. The parameter of uncertainty influenced the carbon model, especially for the parameters of Moso bamboo and clumping bamboo, because the production units of Moso bamboo and clumping bamboo were collected in culms. This study was consistent with previous findings on a national scale. Furthermore, investigations of bamboo end-use in China could address parameter uncertainty to ensure better HBP carbon stock assessment.

## Conclusions

The carbon inflow of HWP/HBP increased over 3 times in 2020 compared to 1987, resulting in a total carbon stock of 458.5 TgC in China. The HWP carbon stock in Heilongjiang peaked in 2010 at 42.0 TgC, making it the highest in the country, but it was surpassed by Guangxi, with 60.8 TgC by the end of 2020. Most provinces showed sustained accumulation of HWP carbon stocks, except for the western provinces (Tibet, Shaanxi, Gansu, Qinghai, and Ningxia) and municipalities (Beijing, Shanghai, Tianjin, and Chongqing). Furthermore, HBP occupied an important place in carbon sequestration in China; 28% of the carbon stock was contributed by HBP in China and Fujian maintained the highest contributor of HBP carbon stock at 33.4 TgC until 2020. Guangxi, Zhejiang, Jiangxi, Yunnan, Sichuan, Chongqing, Guizhou, Jiangsu, Hunan, Anhui, and Guangdong also showed notably increases in the HBP carbon stock from 1992 to 2020. The importance of the HBP carbon stock accumulation exceeded that of the HWP in Fujian, Zhejiang, and Chongqing, with proportions of HBP carbon stock 58.0%, 63.2%, and 72% in 2020, respectively. However, the HBP carbon stocks in Henan, Shaanxi, Hubei, and Hainan declined after 2005. These results indicate large differences in the accumulation of HWP/HBP carbon stocks between provinces in China. The information on provincial-scale carbon stock changes and source proportion between wood and bamboo could be useful in guiding forest management for local policymakers. Particularly, improving the management of harvested bamboo and focusing on the collection of statistical bamboo data is critical for assisting south China in achieving climate mitigation in forestry.

## Data Availability

The datasets generated and analyzed during the current study are available from the corresponding author on reasonable request.
